# Deaminase-based RNA recording enables high throughput mutational profiling of protein–RNA interactions

**DOI:** 10.1101/2025.04.11.648485

**Published:** 2025-04-12

**Authors:** Rachael A. Bakker, Oliver B. Nicholson, Heungwon Park, Arvind Rasi Subramaniam, Christopher P. Lapointe

## Abstract

Protein–RNA interactions govern nearly every aspect of RNA metabolism and are frequently dysregulated in disease. Although individual protein residues and RNA nucleotides critical for these interactions have been characterized, scalable methods that jointly map proteinand RNA-level determinants are limited. RNA deaminase fusions have emerged as a strategy to identify transcriptome-wide targets of RNA-binding proteins by converting binding events into site-specific nucleotide edits. Here, we show that this ‘RNA recording’ approach can be adapted for high-throughput mutational scanning of protein–RNA interfaces. Using the λN–boxB system as a model, we demonstrate that editing by a fused TadA adenosine deaminase correlates with binding affinity between the protein and RNA variants *in vitro* . Systematic variation of RNA sequence context reveals a strong bias for editing at UA dinucleotides by the engineered TadA8.20, mirroring wild-type TadA preferences. We further show that stepwise recruitment of the deaminase using nanobody and protein A/G fusions preserves both sequence and binding specificity. Stable expression of the TadA fusion in human cells recapitulates *in vitro* editing patterns across a library of RNA variants. Finally, comprehensive single amino acid mutagenesis of λN in human cells reveals key residues mediating RNA binding. Together, our results highlight RNA recording as a versatile and scalable tool for dissecting protein–RNA interactions at nucleotide and residue resolution, both *in vitro* and in cells.

## Full Text Availability

The license terms selected by the author(s) for this preprint version do not permit archiving in PMC. The full text is available from the preprint server.

