# Deaminase-based RNA recording enables high throughput mutational profiling of protein-RNA interactions

**DOI:** 10.1101/2025.04.11.648485

**Published:** 2025-04-25

**Authors:** Rachael A. Bakker, Oliver B. Nicholson, Heungwon Park, Yu-Lan Xiao, Weixin Tang, Arvind Rasi Subramaniam, Christopher P. Lapointe

**Affiliations:** 1 Basic Sciences Division, Fred Hutchinson Cancer Center, Seattle, WA 98109, USA; 2 Computational Biology Section of the Public Health Sciences Division, Fred Hutchinson Cancer Center, Seattle, WA 98109, USA; 3 Department of Chemistry and Institute for Biophysical Dynamics, The University of Chicago, Chicago, IL 60637, USA

## Abstract

Protein-RNA interactions govern nearly every aspect of RNA metabolism and are frequently dysregulated in disease. While individual protein residues and RNA nucleotides critical for these interactions have been characterized, scalable methods that jointly map protein- and RNA-level determinants remain limited. RNA deaminase fusions have emerged as a powerful strategy to identify transcriptome-wide targets of RNA-binding proteins by converting binding events into site-specific nucleotide edits. Here, we demonstrate that this ‘RNA recording’ approach enables high-throughput mutational scanning of protein-RNA interfaces. Using the λN-boxB system as a model, we show that editing by a fused TadA adenosine deaminase directly correlates with binding affinity between protein and RNA variants *in vitro*. Systematic variation of RNA sequence context reveals a strong bias for editing at UA dinucleotides by the engineered TadA8.20, mirroring wild-type TadA preferences. We further demonstrate that stepwise recruitment of the deaminase using nanobody and protein A/G fusions maintains both sequence and binding specificity. Stable expression of the TadA fusion in human cells reproduces *in vitro* editing patterns across a library of RNA variants. Finally, comprehensive single amino acid mutagenesis of λN in human cells reveals critical residues mediating RNA binding. Together, our results establish RNA recording as a versatile and scalable tool for dissecting protein-RNA interactions at nucleotide and residue resolution, both *in vitro* and in cells.

## Introduction

RNA-binding proteins (RBPs) play a central role in post-transcriptional gene regulation. They control RNA processing, nuclear export, translation, stability, and subcellular localization. RBPs also mediate the assembly of larger ribonucleoprotein particles and granules, which play roles in diverse cellular functions. Dysregulated RBP-RNA interactions are implicated in a wide range of human diseases (Gebauer et al. 2021). A central goal, therefore, is to identify which RNAs are bound by which RBPs and reveal the molecular bases of the interactions.

Powerful approaches exist to examine RBP-RNA interactions *in vitro* and in cellular contexts, but they have limitations. For example, systematic evolution of ligands by exponential enrichment (SELEX), Bind-N-Seq, and SeqRS examine many thousands of RNA variants bound by an RBP *in vitro* (Tuerk and Gold 1990; Ellington and Szostak 1990; Lambert et al. 2014; Lou et al. 2017; Becker et al. 2019; Jarmoskaite et al. 2019). These strategies define consensus binding motifs across a range of affinities, but they lack physiological context. They also only provide an RNA-centric perspective, as purification of hundreds or thousands of protein variants remains experimentally intractable. Cellular immunoprecipitation-based approaches (e.g., RIP-seq) capture snapshots of RBPs bound to RNAs in more native contexts, but they also may capture non-native interactions that form in lysates (Zhao et al. 2010). Crosslinking strategies, such as CLIP-seq and its many derivatives, circumvent this limitation and provide nucleotide-resolution views of RBP binding in cells (Ule et al. 2005). However, these approaches are limited by crosslinking efficiency, antibody specificity, and biases introduced during crosslinking, RNA isolation, and sequencing. Thus, while current approaches have profoundly advanced our understanding of RBP-RNA interactions, it remains challenging to integrate *in vitro* mutational studies with *in vivo* profiling methods to achieve a more complete understanding of RBP function.

RNA recording-based approaches have emerged as a new strategy to uncover which RNAs are bound by an RBP in cells. In these approaches, an RBP of interest is fused to an RNA modification enzyme. The fusion protein covalently modifies bound RNAs, which can then be quantified by high-throughput sequencing. RNA modifying enzymes used for these studies include a poly(U) polymerase, engineered versions of the adenosine deaminase ADAR, and the cytosine deaminase APOBEC2 (Lapointe et al. 2015; McMahon et al. 2016; Rahman et al. 2018; Meyer 2019; Brannan et al. 2021). More recently, the *E. coli* adenosine deaminase TadA also has been engineered to enhance its efficiency and modify a broad range of RNA substrates (Xiao et al. 2023; Lin et al. 2023). In each case, the resulting fusion proteins identified RNA targets of the RBP that significantly overlapped with ones identified using CLIP-based approaches. The editing marks accumulate in the RNAs over time, require less input material, and can be multiplexed using orthogonal enzymes. Thus, RNA recording provides a broader and complementary view of RBP-RNA interactions compared to direct binding-based approaches.

Given the success of RNA recording in identifying endogenous targets of RBPs, we sought to use this approach for mutational studies of protein-RNA interactions. We reasoned that the extent of editing (i.e., editing efficiency) should correlate with the affinity of an RBP or an RNA variant for its interaction partner. Since RNA editing can be carried out with purified enzymes or in cells, RNA recording can enable direct comparison of protein-RNA interactions between *in vitro* and cellular contexts. Further, expression of RBP variant libraries in cells could be used to study protein-RNA interactions from the protein perspective–a capability that current RNA-centric approaches lack. Here, we test the feasibility of these ideas using a model RBP-RNA system, and thereby demonstrate the utility of RNA recording to map the key molecular determinants of protein-RNA interactions at scale.

## Results

### Recruitment of a deaminase increases its editing efficiency on the RNA target.

**Figure 1:**
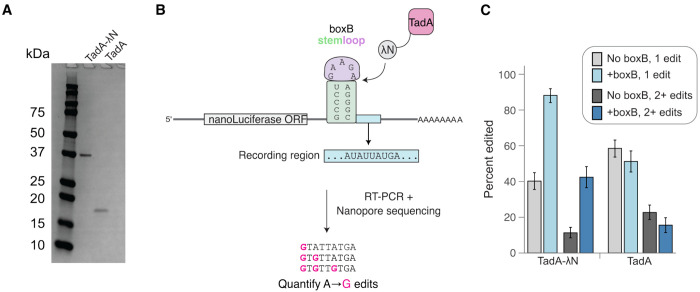
TadA-λN specifically modifies boxB reporter RNA. **A.** SDS-PAGE of purified TadA-λN and free TadA8.20. Proteins visualized with Coomassie stain. **B.** Schematic of boxB stem loop reporter and sequencing strategy to detect A-to-I edits. Control RNA reporters without boxB stem loop are not shown. Elements are not drawn to scale. **C.** Editing efficiency of *in vitro* transcribed reporter RNAs incubated with TadA or TadA-λN. Error bars are 95% confience intervals as determined by a binomial bootstrapping analysis.

We first tested whether fusing a deaminase to an RBP could direct its editing activity to a specific RNA target *in vitro*. As the editor, we used TadA8.20, an evolved variant of the *E. coli* TadA A-to-I deaminase (Wolf et al. 2002). This enzyme has high activity and low sequence specificity on DNA (Gaudelli et al. 2020) and RNA (Xiao et al. 2023). As a model RBP, we selected the 22 amino acid λN peptide that binds to a specific stem loop sequence called boxB (Chattopadhyay et al. 1995), and is widely used to tether proteins to RNAs for *in vivo* functional studies (De Gregorio et al. 1999; Baron-Benhamou et al. 2004). We purified TadA8.20 alone or as a TadA8.20-λN fusion protein (hereafter, TadA-λN) (Figure 1A). We incubated the purified proteins with a model reporter RNA engineered to contain or lack a boxB stem loop (Figure 1B). After incubation, reporter RNAs were reverse transcribed (RT), PCR-amplified, and analyzed by long read nanopore sequencing. A-to-I editing of RNA introduces A-to-G substitutions after RT-PCR, and hence the frequency of A-to-G substitutions in sequenced reads serves as a quantitative measure of editing efficiency (Figure 1B). TadA8.20 alone edited both reporters equally (50–60% editing efficiency, Figure 1C), consistent with its high and non-specific activity (Xiao et al. 2023). In contrast, TadA-λN showed significantly enhanced editing of the boxB-containing RNA (~90%) compared to the boxB-lacking control (40%) (Figure 1C). The increased editing with TadA-λN and boxB-containing RNA was evident in both single-edit or multi-edit analyses. These results demonstrate that recruitment of TadA-λN to boxB-containing RNA targets *in vitro* markedly increased editing efficiency.

### A high throughput editing assay for studying RNA-protein interactions.

**Figure 2:**
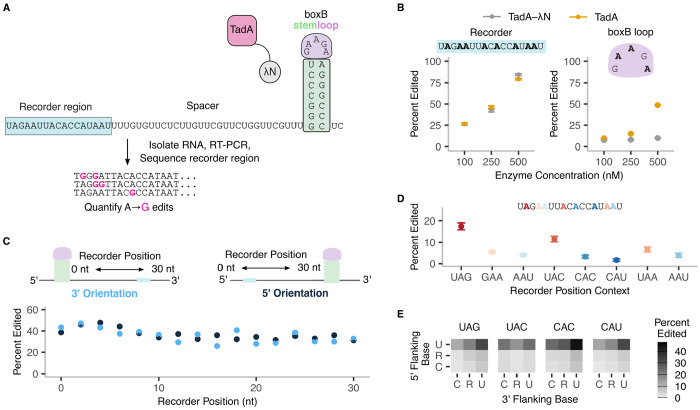
High throughput analysis reveals sequence context dependence of TadA-λN editing. **A.** Schematic of boxB stem loop reporter and high throughput sequencing strategy. **B.** Mean editing efficiency of the recorder region (left) or the boxB loop (right) in the reporters. Error bars represent standard error over technical replicates. **C.** Mean editing efficiency as a function of the distance and orientation between the recorder region and boxB stemloop. Dark blue and light blue points indicate position of the boxB loop at the 5′ or 3′ end of the reporter, respectively. Data corresponds to 250 nM TadA-λN incubated with reporter RNA at 37 °C for 2 hours. Recorder position refers to distance between the 5′-most base of the recorder region and the 5′ end of the reporter RNA. Error bars represent standard error over 2048 recorder sequence variants. **D.** Mean editing efficiency at different adenines within the recorder region. Error bars denote standard error over 24 technical replicates. **E.** Mean editing eficiency as a function of the nucleotide flanking the edited adenine. R represents G and A nucleotides, which were talllied together since we cannot resolve edited As from unedited Gs. Mean is calculated over 30 technical replicates.

To enable mutational studies of RNA-RBP binding at scale, we developed a reporter assay with deep sequencing readout of TadA-mediated RNA editing. Our RNA reporters consisted of a boxB stem loop and an A-rich ‘recorder’ region separated by an A-depleted spacer (Xiao et al. 2023) (Figure 2A). We *in vitro* transcribed the reporter and incubated the purified RNA with either the TadA-λN fusion or TadA8.20 alone. We performed the editing reactions with the enzymes at excess (500nM), equimolar (250nM), or sub-saturating (125nM) concentrations relative to the RNA. After a 2 hour incubation and RT-PCR, we measured the frequency of A-to-G substitutions using Illumina short read sequencing. We examined the editing frequency of the A-rich recorder region and the boxB loop separately. Editing of the recorder region increased similarly at higher enzyme concentrations for both TadA-λN and TadA8.20 (Figure 2B, left). This observation is in line with the high non-specific editing efficiency of TadA8.20 (Xiao et al. 2023). However, while TadA8.20 on its own efficiently edited the adenosines within the boxB loop, TadA-λN editing of the boxB loop was reduced 2–5 fold relative to TadA8.20 at 250nM and 500nM concentrations (Figure 2B, right). This observation is consistent with TadA-λN binding the boxB loop and protecting the adenosines within it from editing.

As a proof-of-principle pooled experiment, we examined whether the distance between and orientation of the boxB stem loop and the recorder region affected editing efficiency of the reporter. We synthesized a pooled RNA library with varying distances (0–30nt) between the boxB stem loop and the recorder region in either 5′ and 3′ orientations (Figure 2C, top). We incubated the library with TadA-λN and sequenced the edited regions as above. TadA-λN edited recorder regions at various distances from boxB at similar efficiencies (Figure 2C). This lack of distance and orientation preference might arise from the long flexible linker (96aa) between the λN and TadA domains in our construct. Thus, for all subsequent pooled library experiments, we included both recorder region orientations and combined the data for analyses.

### TadA8.20 editing is sensitive to the sequence context of the edited adenosine.

TadA8.20 was evolved from a natural *E. coli* enzyme that edits the adenosine within a UAC loop in a specific tRNA (Wolf et al. 2002). Since engineered TadA enzymes also exhibit editing preference towards adenosines adjacent to pyrimidines (T or C) on DNA (Gaudelli et al. 2020; Xiao et al. 2024b), we examined whether Tad8.20 might exhibit sequence context preferences during RNA editing. We analyzed editing across the 8 adenosines of the recorder region in our reporters, each of which has a unique combination of 5′ and 3′ flanking bases. The UAG and UAC contexts had the highest editing rates, at 17.5 % and 11.6 % respectively (Figure 2D), with the latter context the same as the tRNA sequence context of the natural TadA enzyme. The six other adenosines in our recorder region had 2- to 10-fold lower editing efficiency relative to UAG. We observed these differences between adenosine contexts at all concentrations of TadA-λN tested (Supplementary Figure 1A).

To assess the apparent sequence bias of TadA8.20 more systematically, we designed a library with randomized sequence contexts around the eight adenosines in the recorder region of our reporter (Figure 2A). This randomization yielded four adenosines with all possible combinations of 5′ and 3′ flanking nucleotides, and four other adenosines with only the 5′ or the 3′ flanking nucleotide varied. For analyzing the results, we combined flanking adenosines and guanosines into a single purine base ‘R’, since edited adenosines are indistinguishable from unmodified guanosines. Consistent with our analysis of the unmodified recorder region, all adenosines with a 5′ uridine were edited at 5- to 10-fold higher rates than other sequence combinations (Figure 2E, Supplementary Figure 1B). Presence of a 3′ U (UAU context) further enhanced editing by 1.5–2 fold, and resulted in the highest editing efficiency across all flanking contexts at 24–45%. Conversely, the CAC or CAR flanking contexts had the lowest editing efficiency at 1–2%. Together, our analyses show that TadA8.20, despite its high editing efficiency on RNA, retains its native specificity for UA dinucleotide motifs (Wolf et al. 2002).

We also compared our *in vitro* results to another TadA-derived RNA base editor, rABE, that was recently used to identify RBP binding sites *in vivo* (Lin et al. 2023). In that published data, we found that the TadA7.10-derived rABE base editor had higher editing efficiency when the edited adenosine was flanked by a 5′ U or C (Supplementary Figure 1C). This observation on native RNAs is consistent with TadA7.10’s preference on DNA (Xiao et al. 2024b). By contrast, our findings demonstrate that TadA8.20 exhibits a preference for only UA, suggesting that the two enzyme variants have distinct sequence preferences on RNA.

### TadA-λN editing quantitatively reflects RNA-RBP binding strength *in vitro*.

**Figure 3:**
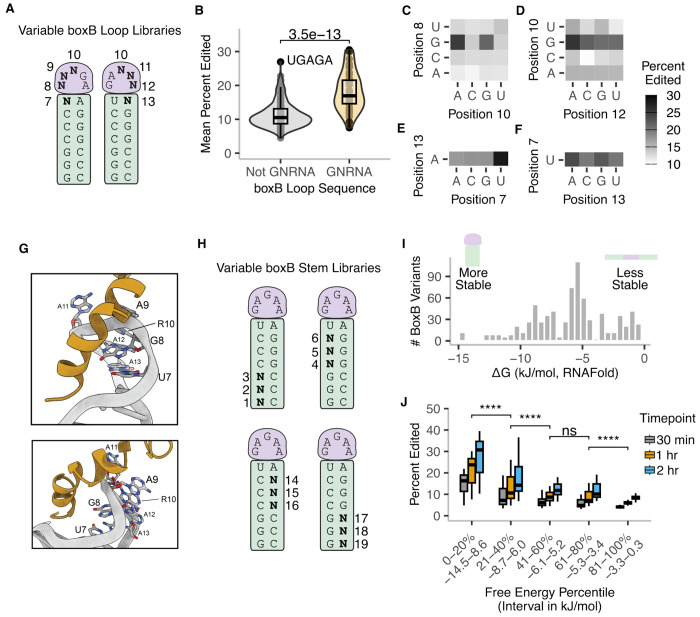
TadA-λN editing quantitatively reflects RNA-RBP binding strength *in vitro*. **A.** boxB loop variant library design. N indicates a randomized base. **B.** Mean editing efficiency of boxB loop variants with GNRNA motifs (n=41) to those without (n=223). Box plots indicate median and inter-quartile ranges. P-values calculated using two-sided Wilcoxon test. **C-F.** Mean editing efficiency as a function of nucleotide at location 8 and 10 (C), 10 and 12 (D), 7 (E), 13 (F) of the boxB loop. **G.** NMR structure of λN peptide boxB complex with labeled loop nucleotides (Schärpf et al. 2000). λN is colored gold. **H.** boxB stem variant library design. N indicates a randomized base. **I.** Distribution of estimated free energy of all boxB stem variants. Free energy was calculated using RNAFold within the ViennaRNA package (Lorenz et al. 2011). **J.** Mean editing efficiency of boxB stem variants. The 256 boxB stem variants were divided into 5 quintiles. Box plots indicate median and inter-quartile ranges. P-values were calculated using two-sided Wilcoxon test. **** p < 0.0001, n.s. p > 0.05.

*In vivo* expression of deaminase-RBP fusions yields variable editing efficiencies across endogenous RNAs (Medina-Munoz et al. 2024). However, because endogenous RNAs differ in sequence, structure, and associated RBPs, it is unclear whether editing efficiency reliably reflects RBP binding strength. To directly test this relationship, we used our *in vitro* system to measure editing across defined RNA libraries with controlled sequence variation. We designed reporter libraries in which the boxB stem and loop regions were randomized in 3nt or 4nt windows, while the recorder region remained constant (Figure 3A,H; Supplementary Table 1). We incubated these libraries with either sub-saturating or saturating concentrations of TadA-λN for varying durations, and quantified the average A-to-G substitution frequency for each boxB variant.

Editing by λN–TadA recapitulated several known features required for λN binding to boxB (Figure 3B–H). Prior structural and biochemical analyses showed that λN preferentially binds a GNRNA pentaloop (N=A/C/G/U, R=A/G) (Legault et al. 1998). This sequence forms a GNRA tetraloop (with the second N extruded), a common RNA fold recognized by many RBPs (Thapar et al. 2014). Consistently, boxB variants containing a GNRNA motif in its loop were edited more efficiently by TadA-λN than those without under sub-saturating enzyme concentrations (median editing: 15 vs 10%, p=3.5e-13) (Figure 3B). The GAAGA motif from wild-type boxB ranked among the highest-edited sequences (30.1%, Figure 3B). Interestingly, a non-canonical variant (UGAGA) was also highly edited (Figure 3B), suggesting that λN can tolerate alternative sequence registers in boxB that may adopt similar RNA folds. Further analysis revealed that guanosine at position 8 and a purine at position 10—core components of the GNRNA motif—were associated with the highest editing levels (Figure 3C). These findings agree with prior evidence that G8 and A10 are required for high-affinity λN binding and transcriptional regulation (Chattopadhyay et al. 1995; Tan and Frankel 1995). Position 12 is part of the GNRNA tetraloop, but it does not directly contact λN residues in structures of the λN-boxB complex (Legault et al. 1998; Schärpf et al. 2000). An A in position 12 yielded moderately higher editing (~0.8–fold) when position 10 was G, and showed a preference for R when position 10 was a pyrimidine (Figure 3D). This is consistent with position 12 playing a secondary role in λN recognition of boxB, likely by stabilizing the tetraloop structure. These results reinforce the importance of GNRA-like motifs for λN binding to boxB.

In addition to the loop, λN requires the closing U7-A13 basepair of the stem for high-affinity binding (Tan and Frankel 1995). Variants preserving this base pair exhibited higher editing rates than mismatched pairs (Figures 3E,F). Uridine at position 7–known to make direct contats with λN in structures (Schärpf et al. 2000)–was strongly enriched for higher editing (Figure 3G). In contrast, adenosine at position 13 was only weakly favored, and all four nucleotides supported relatively high levels of editing (Figure 3F). These observations suggest that U7 is the key determinant, while base pairing at this position contributes less. At saturating concentration of TadA-λN, enrichment for U7 and the GNRNA-like motifs diminished, suggesting increased non-specific editing, as expected (Supplementary Figure 2A). Consistently, TadA8.20 alone did not reproduce these features for high-affinity λN binding (Supplementary Figure 2B).

Editing efficiency also strongly correlated with the thermodynamic stability of the boxB stem. We used RNAfold (Lorenz et al. 2011) to calculate the predicted free energy (ΔG) of each stem variant in our libraries (Figure 3I), and grouped them into bins from most to least stable. On average, TadA-λN more efficiently edited boxB variants predicted to have more stable boxB stems than those with less stable stems (Figure 3J). This trend was evident across timepoints, with editing efficiency increasing over time. While diminished relative to TadA-λN, TadA8.20 alone also showed modestly higher editing for the most stable stem variants (Supplementary Figure 2C, bottom), suggesting that TadA8.20 itself may bind RNA hairpins at low levels, contributing to off-target effects. Increasing the concentration of TadA-λN or TadA8.20 to a saturating level also decreased the correlation between editing efficiency and apparent stem stability, consistent with the expected shift to a non-specific binding regime (Supplementary Figure 2C,right). Together, these results demonstrate that TadA-λN editing quantitatively reflects RNA–RBP binding strength *in vitro*, capturing both sequence and structural determinants of high-affinity λN–boxB recognition.

### Split recruitment preserve RNA editing specificity.

**Figure 4:**
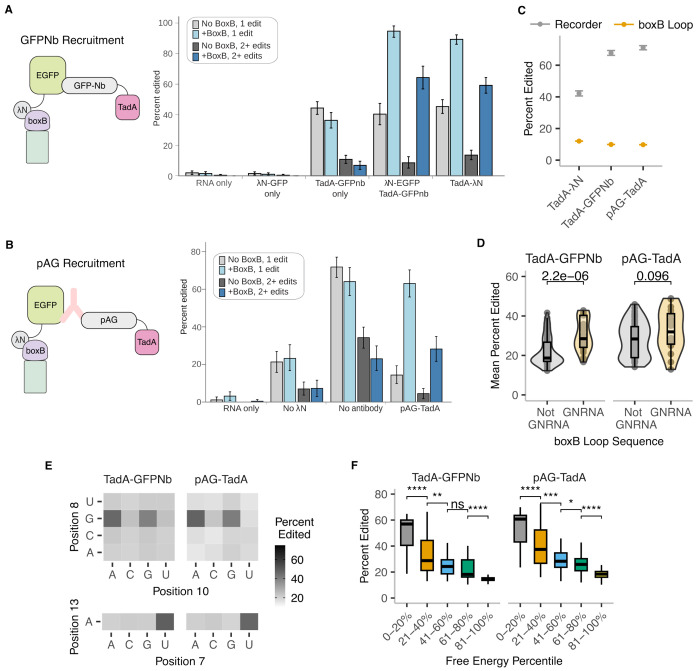
Split recruitment of TadA and λN preserves RNA editing specificity. **A.** (Left) Schematic of TadA-GFPNb recruitment strategy to boxB RNA reporters. (Right) Editing efficiency of *in vitro* transcribed nanoluciferase reporter RNAs incubated with indicated components (see diagram, Figure 1B). Error bars are 95% confience intervals as determined by a binomial bootstrapping analysis. **B.** (Left) Schematic of pAG-TadA recruitment strategy to boxB RNA reporters. (Right) Editing efficiency of *in vitro* transcribed nanoluciferase reporter RNAs incubated with indicated components (see diagram, Figure 1B). Error bars are 95% confience intervals as determined by a binomial bootstrapping analysis. **C.** Mean editing efficiency across different recruitment methods in either the recorder region (grey) or boxB loop (orange). Error bars represent standard error over n=64 technical replicates. TadA-λN data same as Figure 2, included here for comparison. **D.** Comparison of editing efficiency of boxB loop variants with GNRNA motifs (n=26 for TadA-GFPNb, n=18 for pAG-TadA) to those without (n=126 for TadA-GFPNb and n=61 for pAG-TadA). Box plots indicate median and inter-quartile ranges. P-values calculated using two-sided Wilcoxon test. **E.** Mean editing efficiency as a function of nucleotide identity at location 8 and 10 (top), and base 7 (bottom) of the boxB loop. **F.** Mean editing efficiency of boxB stem variants. Free energy intervals are identical to those indicated in Figure 2J x-axis. Box plots indicate median and inter-quartile ranges. P-values were calculated using two-sided Wilcoxon test. **** p < 0.0001, *** p < 0.001, ** p < 0.01, * p < 0.05, n.s p > 0.05.

In addition to direct fusion of RBPs to RNA-modifying enzymes, recent studies have used nanobody and protein A/G fusions to recruit RNA editors and reverse transcriptases to RBPs (Liang et al. 2024; Xiao et al. 2024a; Khyzha et al. 2022). These “split” strategies eliminate the need to generate deaminase fusions for each RBP, enabling broader application to fixed cell lines and tissues. However, it remains unclear how the efficiencies and specificities of split recruitment approaches compare to those of the direct fusion approach. To address this question, we examined two split recruitment strategies using a nanobody or protein A/G (pAG) to recruit TadA8.20 to boxB-containing RNAs. To enable direct comparison, we purified λN-GFP and used either a purified TadA8.20-GFP nanobody fusion (henceforth TadA-GFPnb) or a monoclonal anti-GFP antibody in combination with purified pAG-TadA8.20 (henceforth pAG-TadA) (Figure 4A,B, Supplementary Figure 3A).

Reporter mRNAs with or without a boxB stem loop were incubated with λN-GFP and either TadA-GFPnb or the anti-GFP primary antibody and pAG-TadA proteins, followed by RT-PCR and nanopore sequencing. Incubation with TadA-GFPnb or pAG-TadA alone (without λN-GFP or anti-GFP antibody present) edited both reporter mRNAs regardless of boxB sequence (Figure 4B), similar to background editing observed with the direct TadA-λN fusion. By contrast, addition of λN-GFP yielded a 2–4 fold increase in editing of the boxB-containing mRNA relative to boxB-lacking mRNA in both split recruitment strategies (Figure 4B). Using our high-throughput reporter assay, we found that both split recruitment strategies produced robust editing in the recorder region, whereas the boxB loop sequence was edited at a lower frequency (Figure 4C). Both TadA-GFPnb and pAG-TadA retained their preference for UA dinucleotides in the recorder region (Supplementary Figure 3B). Together, these results show that both split recruitment strategies recapitulate the editing specificity of the direct TadA-λN fusion.

We tested the split recruitment strategies on the boxB loop- and stem-randomized libraries to determine if they recover sequence and structural preferences of λN binding. Both split strategies yielded higher editing when recruited by GNRNA boxB loops relative to non-GNRNA loops (Figure 4D). However, several non-GNRNA loops exhibited comparable editing to GNRNA loops when recruiting pAG-TadA. This finding suggests that the increased complexity of this strategy, requiring successful formation of a four component complex, may reduce specificity. Nevertheless, both split strategies showed higher editing when recruited by loop variants with a G in position 8, a purine in position 10, and a uridine in position 7 (Figure 4E), while position 12 had minimal influence (Supplementary Figure 3C). Editing efficiency of both approaches also correlated with the predicted stability of the boxB stems (Figure 4F). Finally, the extent of editing by TadA-GFPnb or pAG-TadA significantly correlated with that of TadA-λN, but diverged from that of TadA8.20 alone (Supplementary Figure 3D–E). Together, these results show that the two split recruitment strategies preserve key hallmarks of high-affinity binding by λN, but can result in higher non-specific editing than the direct fusion approach in certain contexts.

### *In vivo* analysis of TadA-λN recruitment and editing

**Figure 5:**
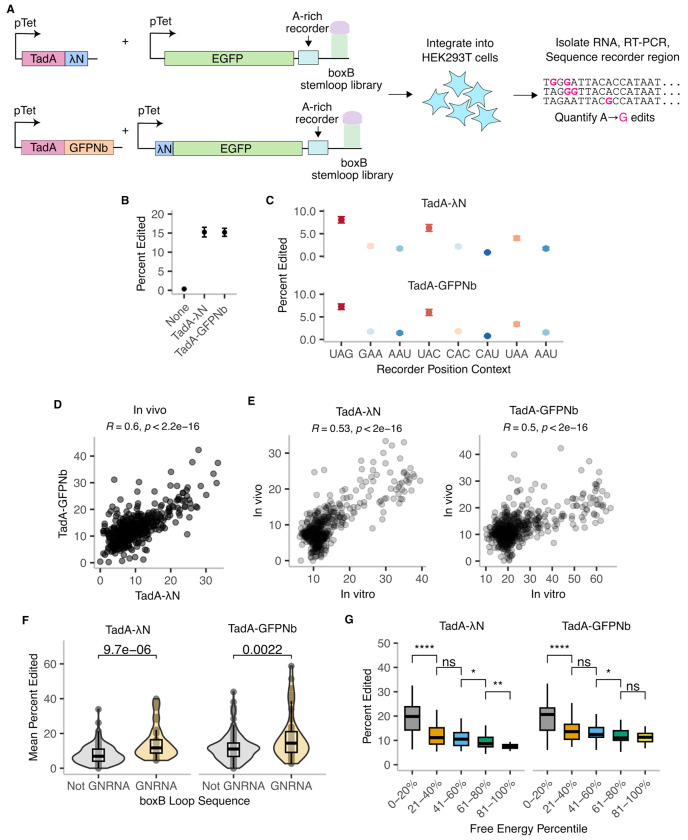
*In vivo* TadA-λN editing reflects binding strength and context preferences. **A.** Schematic of TadA and boxB libraries design and integration into HEK293T cells. Elements not drawn to scale. **B.** Mean editing efficiency in each *in vivo* library recorder region for wild-type BoxB reporters. Error bars represent standard error over n=24 technical replicates. **C.** Mean editing efficiency at different adenines within the recorder region. Error bars denote standard error over 12 technical replicates. **D.** Comparison of editing efficiency between TadA-GFPNb and TadA-λN *in vivo* for individual boxB variants. R represents Spearman correlation coefficient. **E.** Comparison of editing efficiency between *in vitro* and *in vivo* for boxB variants in cells expressing TadA-λN or TadA-GFPNb. R represents Spearman correlation coefficient. **F.** Comparison of editing efficiency of boxB loop variants with GNRNA motifs (n=33 for TadA-LN and n=42 for TadA-GFPNb) to those without (n= 141 for TadA-LN and n=208 for TadA-GFPNb). Box plots indicate median and inter-quartile ranges. P-values calculated using two-sided Wilcoxon test. **G** Mean editing efficiency of boxB stem variants. Free energy intervals are identical to those indicated in Figure 2J x-axis. Box plots indicate median and inter-quartile ranges. P-values calculated using two-sided Wilcoxon test. **** p < 0.0001, *** p < 0.001, ** p < 0.01, * p < 0.05, n.s p > 0.05.

We next asked whether editing patterns observed *in vitro* with purified enzymes are preserved when the same constructs are expressed *in vivo* in human cells. We focused on editing by TadA-λN and TadA-GFPNb for our *in vivo* experiments, as pAG-TadA requires antibody binding and is not readily applicable to living cells. We designed a reporter library consisting of either EGFP or λN-EGFP mRNA with a boxB stem loop and an A-rich recorder region in the 3′ UTR (Figure 5A). The boxB stem loop was randomized in 3- or 4-nucleotide increments similar to our previous *in vitro* stem loop libraries. We co-expressed the EGFP and λN-EGFP reporter libraries with either TadA-λN or TadA-GFPNb, with both the reporter and the TadA constructs under the control of a doxycycline-inducible promoter. We integrated the libraries into the *AAVS1* locus of HEK293T cells using site-specific Bxb1-mediated integration (Nugent et al. 2024), ensuring that each cell expressed a single boxB variant in combination with either TadA-λN or TadA-GFPNb. We also generated a control cell line expressing only the EGFP reporter, without a TadA construct. After doxycycline induction for 72 hours, we harvested RNA and analyzed editing efficiency by deep sequencing the 3′ UTR of the reporter (Figure 5A).

Cells expressing TadA-λN or TadA-GFPnb showed increased editing in the recorder region compared to control cells not expressing TadA (15% vs 0.3% reads with 1 or more edit, Figure 5B). We observed higher editing in the UAG, UAC, and UAA sequence contexts relative to the other trinucleotide contexts in the recorder region (Figure 5C), mirroring our *in vitro* observations. Editing rates were lower *in vivo* than *in vitro* at all concentrations, presumably due to limiting *in vivo* enzyme levels arising from single copy integration. Editing rates for different boxB variants were correlated between the direct fusion and split recruitment strategies *in vivo* (R=0.5) (Figure 5D). Notably, the *in vivo* editing rates of the boxB variants were also significantly correlated with the *in vitro* editing rates (Figure 5E). The *in vivo* correlation was slightly stronger for the direct fusion than for split recruitment (R=0.53 vs R=0.5), presumably reflecting the more complex requirement in the latter case for two proteins and the RNA reporter to bind together in the crowded cellular environment. For both the direct fusion and the split recruitment strategies, GNRNA boxB loop variants resulted in significantly higher editing rates than non-GNRNA variants (Figure 5F), confirming that TadA-λN binding specificity observed *in vitro* persists in cells. Given the overall lower levels of editing, comparisons of base combinations at different positions were noisy, though we observed consistently elevated editing levels for guanosine at positions 8, 10 and 12 (Supplementary Figure 4A and B). While differences in editing based on stem stability were less pronounced in the cellular context compared to *in vitro* results, stronger hairpins still exhibited higher editing rates, with the strongest hairpins showing a significantly elevated editing (Figure 5G). RNA secondary structures are subject to additional layers of regulation in a cellular context due to interactions with intracellular RBPs (Georgakopoulos-Soares et al. 2022), which may explain why calculated free energy is less predictive of λN binding-mediated RNA editing. In summary, TadA-λN editing patterns in cells recapitulate our *in vitro* results, albeit with reduced resolution across boxB variants and overall lower editing levels.

### Deep mutational scanning of the λN RNA-binding domain

**Figure 6:**
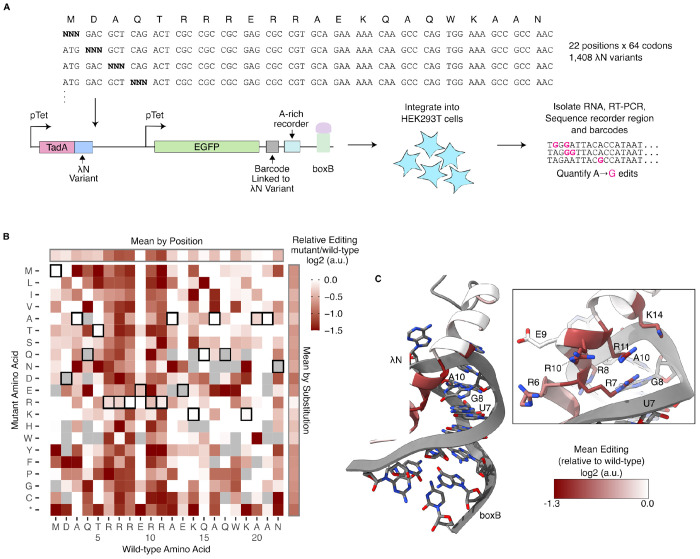
Deep mutational scanning of λN reveals key residues mediating RNA binding. **A.** Schematic of DMS library design and integration strategy into HEK293T cells. Elements not drawn to scale. **B.** Relative editing efficiency of λN variants as a function of residue position and identity. Log2 ratios of mutant to wildtype are plotted from red (>−1.5, loss of editing) to white (>0, neutral or gain of editing). Grey boxes indicates <3 barcodes were recovered for that amino acid variant. Wild-type residues are indicated by a black outline. **C.** Per-residue log2 ratio of normalized mean change in editing for nonsynonymous mutations mapped onto NMR structure for boxB-N peptide complex (Schärpf et al. 2000).

Since the above *in vivo* experiments resolved affinity differences between an invariant λN and boxB RNA variants, we next asked whether RNA editing can also be used to study interactions between a fixed boxB stemloop and λN peptide variants. Such a high-throughput approach would complement existing methods that probe RNA variant libraries against fixed RBPs (Tuerk and Gold 1990; Ellington and Szostak 1990; Lambert et al. 2014; Lou et al. 2017). To this end, we constructed a comprehensive single-codon substitution library by randomizing all 22 codons of the λN open reading frame, yielding 1,408 (22 × 64) unique variants. We expressed this λN variant library as a fusion with TadA8.20 and co-expressed it with an mRNA reporter containing a boxB stem loop and an A-rich recorder region in the 3′ UTR (Figure 6A). Both the TadA-λN and reporter expression cassettes were under the control of a doxycline-inducible promoter as in our previous *in vivo* experiment. We included a random 20-nucleotide A-depleted barcode upstream of the recorder region during cloning, allowing each λN codon variant to be uniquely linked to a median of 8 barcodes, as confirmed by deep sequencing (Supplementary Figure 5A). We integrated the libraries into the *AAVS1* locus of HEK293T cells using site-specific Bxb1-mediated integration (Nugent et al. 2024), ensuring that each cell expressed a single λN codon variant. After 72 hours of doxycycline induction, we harvested RNA and deep sequenced the 3′UTR to measure editing efficiency in the recorder region, and assigned each read to a λN variant via its associated barcode.

While the editing efficiency by individual λN amino acid variants was noisy (Supplementary Figure 5A), several biologically meaningful patterns emerged (Figure 6B). First, mutations introducing premature stop codons resulted in the largest decrease in editing efficiency, consistent with disruption of λN–boxB binding by truncated peptides (Figure 6B). Second, nearly all substitutions of wild-type arginine codons at positions 6, 7, 8, 10, and 11 led to substantial reductions in editing (Figure 6B). Comparison with an an existing NMR structure for the λN-boxB complex (Schärpf et al. 2000) revealed that these positions map to the face of the α-helix that directly contacts the boxB hairpin (Figure 6C). This is consistent with previous biochemical studies showing that the arginine-rich α-helical motif of λN is essential for both boxB recognition and helix stabilization (Chattopadhyay et al. 1995; Tan and Frankel 1995). In particular, Arg7 and Arg11–both of which make close contacts with nucleotide U7 of boxB–exhibited the lowest mean editing efficiencies (Figure 6C, right panel). Glu9 was the only residue within the arginine-rich motif whose mutation had minimal effect on editing effiency, consistent with its orientation away from the RNA interface and lack of direct contacts with boxB. Substituions at Lys14 also reduced editing in many cases, likely reflecting its proximity to the RNA backbone (Figure 6C, right panel). Together, these results show that *in vivo* RNA recording can be effectively combined with deep mutational scanning to identify amino acid residues in RBPs that are critical for RNA recognition and binding.

## Discussion

Here, we present a high-throughput strategy to interrogate the molecular interactions that underlie protein–RNA binding. Our RNA recording strategy leverages an RNA editing approach commonly used to map transcriptome-wide RBP binding sites. We adapt this system to comprehensively assess the effects of amino acid and nucleotide mutations on RBP–RNA interactions. We find that deamination by an RNA editor fused to an RBP captures changes in RNA–RBP interactions both *in vitro* and in cells. We show that this strategy, which relies on high throughput DNA sequencing to measure RNA editing, can be applied across diverse libraries of sequence variants, with mutations introduced on either the RNA or the protein side.

Our RNA recording system used an engineered adenosine deaminase, TadA8.20, derived from a natural tRNA deaminase from *E. coli*.(Wolf et al. 2002) Given its high activity on nucleic acid substrates, TadA8.20 is well suited for RNA recording applications (Gaudelli et al. 2020). Indeed, we confirmed that TadA8.20 deaminates adenosines in a variety of sequence contexts both *in vitro* and in cells. However, by systematically varying the adenosine context, we find that TadA8.20 partly retains the substrate specificity of its tRNA-deaminating ancestor, which targets A34 flanked by U33 and C35 in the anticodon loop of a tRNA(Wolf et al. 2002). Specifically, the identity of the nucleotide immediately preceding the adenosine strongly impacted the editing efficiency. TadA8.20 preferentially deaminated UA dinucleotides, with up to 10-fold higher activity than for other dinucleotide motifs. We also found that the rABE editor–a distinct TadA variant with two substitutions relative to TadA8.20–exhibits similar but distinct preferences, favoring UA and CA motifs in cells (Lin et al. 2023). Thus, recent efforts (Xiao et al. 2024b) to broaden the editing context of TadA by reducing its DNA specificity may further improve its utility for RNA recording applications.

Our RNA recording strategy captured several key determinants of high-affinity binding between the RNA-binding domain of λN and its cognate boxB RNA target. Using libraries of boxB RNA variants, we found that substitution of U7–which pairs with A13 to close the boxB stem–and G8–the first nucleotide of the boxB loop–led to the largest decreases in editing efficiency *in vitro* and in human cells. Consistently, both nucleotides make direct contacts with λN in structures of the complex (Legault et al. 1998; Schärpf et al. 2000). Substitution of three other nucleotides within the loop (A9, G11, A12) and A13 of the closing base pair had intermediate or minimal effects in our assays. These bases lack direct contacts with λN in the structure (Legault et al. 1998; Schärpf et al. 2000). Conversely, substitution of A10, the third loop position, to guanosine had little effect, while substitution to a pyrimidine dramatically impaired λN binding. This position forms the purine core of the GNRNA tetraloop required for the boxB hairpin to adopt its functional conformation. From the protein side, our deep mutational scanning of >400 λN variants identified the arginine-rich patch spanning residues 6–11 as most critical for RNA editing in human cells. Conversely, substitution of Glu9, an internal residue surrounded by the arginine patch, was largely inert, consistent with its lack of direct RNA contacts and prior studies. Thus, RNA recording can pinpoint individual nucleotide or amino acid residues that are essential or dispensable for an RBP-RNA interaction.

While we demonstrated our approach using the λN–boxB system, RNA recording could be extended to a wide range of RBPs and biological contexts. Prior *in vivo* strategies to map protein–RNA interactions or mutation effects often relied on genetic, transcriptional, or reporter-based readouts (SenGupta et al. 1996; Melamed et al. 2013). By using RNA edits as a molecular proxy of binding, RNA recording enables more direct and scalable mutational dissection of RBP–RNA interactions in human cells. Furthermore, we show that TadA8.20 recruitment via direct fusion, nanobody-based tethering, or antibody-pA/G tethering discriminates between high- and low-affinity binding events. This flexibility will enable adaptation of the method to purified systems, live cells, or fixed tissues. However, our findings also highlight trade-offs: increased complexity in recruitment strategies can reduce editing efficiency and resolution. Optimizing the ratios and delivery of each component may be especially important in complex or heterogeneous biological samples. Nonetheless, our work establishes RNA recording with deaminase fusions as a versatile, high throughput platform for identifying the molecular determinants of protein–RNA interaction.

## Materials and Methods

Plasmids, oligonucleotides, and cell lines used in this study are listed in supplemental tables S1–S3. DNA sequences of plasmids used in this study are available at https://github.com/rasilab/bakker_2025/. Information not included below can be requested at https://github.com/rasilab/bakker_2025/issues/.

### Plasmid construction

#### Protein expression plasmids:

The parent TadA8.20 expression plasmid in a pET28a backbone was described previously (Xiao et al. 2023). To generate TadA-GFPNb expression vector (pAS95), Gibson assembly was performed using this plasmid as a backbone along with the following components: a His14x-Avi-SumoEu1 fragment (amplified with primers oRB96 and oRB97), a 48-amino-acid extended XTEN linker (Yarnall et al. 2023), and the GFPNb sequence. This vector (pAS95) served as the template for constructing additional protein expression plasmids.

To generate the TadA-λN expression plasmid (pAS335), pAS95 was digested with BamHI and XhoI to remove the GFPNb insert. The resulting backbone was assembled via Gibson with a synthetic λN fragment (oAS2160, ordered from GenScript).

To construct the pAG-TadA expression plasmid (pAS428), pAS95 was digested with XhoI and SacI to remove the XTEN-GFPNb region. The backbone was assembled with the pAG sequence (amplified from the pAG/MNase plasmid, Addgene #123461, using primers oRB226, oRB227, and oRB228; a gift from the Henikoff lab) and a pAG-specific linker (amplified from pAG/MNase using primers oRB231 and oRB242).

To generate the λN-EGFP expression plasmid, pAS95 was digested with BamHI and XhoI to remove the TadA-GFPNb insert. The resulting backbone was assembled with a synthetic λN fragment (oAS2159, ordered from GenScript) and an EGFP-containing sequence.

#### Reporter libraries for *in vivo* expression:

First, TadA-GFPNb and TadA-λN coding sequences were amplified from their respective expression plasmids using primers oRB245/oRB246 and oRB247/oRB248, respectively. These fragments were cloned using Gibson assembly into a backbone vector containing a cHS4 insulator sequence and pTet Doxycycline inducible promoter sequence. These plasmids were pAS440 (TadA-GFPNb) and pAS441 (TadA-λN).

Next, the resulting plasmids were digested with *MluI* and *AgeI*, and new constructs were assembled using a fragment containing the rbGlobin_pA polyadenylation signal, a pTet promoter, and either the EGFP or λN-EGFP coding sequence (amplified with ) to create pAS443 (TadA-GFPNb + λN-EGFP) and pAS444 (TadA-λN + EGFP). These promoter-EGFP plasmids were then digested with *NotI* and ligated into EcoRV-digested parent vectors (pAS457) containing attB sequences for Bxb1 recombinase integration into the genome, mCherry, and puromycin resistance as markers for integration. The resulting intermediate vectors were pAS472(TadA-GFPNb) and pAS473 (TadA-λN). Following this, these intermediate vectors were digested with *NotI*, and the boxB reporter library oligo pool (oRB262) was inserted via Gibson assembly to create pAS475 (TadA-eGFP) and pAS476 (TadA-λN). The resulting library was cloned with >300,000 colonies to retain library complexity.

To construct the λN site saturation mutagenesis library, the parent vector pAS457 was digested with *MluI* and *SpeI* to remove the TadA-λN insert. This was replaced with a TadA-XTEN fragment (amplified with oRB276/oRB283) via Gibson assembly. In parallel, the boxB reporter sequence was amplified with oRB268/oRB269 and cloned into a separate plasmid bearing the attB site using the NEBuilder HiFi DNA Assembly system (NEB). These two intermediate plasmids were then digested with NotI and EcoRV, respectively, and assembled via Gibson to generate pAS496.

The λN site saturation mutagenesis library was synthesized as an oligo pool (oRB275) by IDT. This pool was amplified using primers oRB271 and oRB284 to append a unique 20-nt DNA barcode and homology arms for Gibson assembly. The plasmid pAS496 was digested with *AgeI* and *MluI*, and the barcoded λN mutagenesis library was inserted in-frame with the upstream TadA-XTEN via Gibson assembly. The final transformants were bottlenecked to 17,500 colonies, providing >10× coverage of the 1,408 λN variants in the library. The resulting plasmid pool (pAS499) was sequenced to link each 20-nt barcode to its corresponding λN variant.

To complete the functional reporter construct, pAS499 was digested with *MluI*, and a fragment containing the rbGlobin terminator, pTet promoter, and EGFP coding sequence (from pAS444 digested with AgeI and MluI) was inserted via Gibson assembly. This positioned the barcode and boxB reporter within the 3′ UTR of EGFP. The final library was cloned with >2 million colonies to maintain high representation of λN variants and barcodes. The resulting plasmid pool (pAS517) was used for genomic integration into cell lines.

### Protein purification

#### λN-EGFP:

The plasmid was transformed into Rosetta 2 cells purchased from the UC Berkeley QB3 MacroLab and grown overnight at 37 °C on LB agar plates supplemented with 50 *μ*g/mL of kanamycin. Liquid cultures of single colonies were grown at 37 °C in LB supplemented with kanamycin. At an OD_600_ of 0.5, isopropyl β-D-1-thiogalactopyranoside (IPTG) was added at a final concentration of 0.5 mM. After two hours at 37 °C, the cultures were shifted to 16 °C and grown overnight. Cells were harvested by centrifugation at 5,000 x g for 10 minutes at 4 °C in a Fiberlite F9 rotor (ThermoFisher, cat # 096-061075). Cells were lysed by sonication in lysis buffer (20 mM Tris-HCl pH 8.0, 300 mM NaCl, 10% (v/v) glycerol, 30 mM imidazole, and 5 mM β-mercaptoethanol) supplemented with protease inhibitors (ThermoFisher, cat # A32965). Lysates were cleared by centrifugation at 27,000 x g for 45 minutes at 4 °C in a Sorvall SS-34 rotor. Clarified lysate was loaded onto Ni-NTA resin (Qiagen, cat # 30210) equilibrated in lysis buffer in a gravity flow column. The resin was then washed with 10 column volumes (CV) of lysis buffer, 10 CV of wash buffer (20 mM Tris-HCl pH 8.0, 1000 mM NaCl, 10% (v/v) glycerol, 30 mM imidazole, and 5 mM β-mercaptoethanol), and 10 CV of lysis buffer. Recombinant protein was eluted in five CV of elution buffer (20 mM Tris-HCl pH 8.0, 300 mM NaCl, 10% (v/v) glycerol, 300 mM imidazole, and 5 mM β-mercaptoethanol). Fractions with recombinant protein were identified by SDS-PAGE analysis. Relevant fractions were dialyzed overnight at 4 °C into dialysis buffer (20 mM Tris Ph 8.0, 250 mM NaCl, 10% (v/v) glycerol, and 5 mM β-mercaptoethanol) in the presence of TEV protease. TEV protease and the cleaved 14xHis-SUMO-Avi tag were captured via a subtractive Ni-NTA gravity column equilibrated in dialysis buffer supplemented with 30 mM imidazole. The flowthrough was collected and concentrated to 500 *μ*L with a 10k MWCO concentrator (MilliporeSigma, cat # UFC901096) then applied to a 23 mL Superdex 200 Increase 10/300 GL SEC column (Cytiva, cat # 28990944). Fractions that contained purified λN-EGFP were concentrated, frozen in liquid N2, and stored at −80 °C.

#### TadA-GFPnb:

Protein was purified as described above with the following modifications. Following the subtractive Ni-NTA step, the protein was diluted to 75 mM NaCl using 20 mM Tris-HCl pH 7.5 and applied to a 1 mL heparin column (Cytiva, cat# 17040601). Purified protein was eluted using a 50 to 1000 mM NaCl gradient in the absence of reducing agents. Fractions that contained purified λN-EGFP were concentrated, frozen in liquid N2, and stored at −80 °C.

#### TadA-λN:

Protein was purified as described above for TadA-GFPnb.

#### pAG-TadA-ybbR:

Protein was purified as described above for TadA-GFPnb. Fractions with recombinant protein from the 1 mL heparin column were collected, concentrated, and further purified using a SD200 120 mL SEC column (Cytiva, cat # 28989335). Fractions that contained purified λN-eGFP were concentrated, frozen in liquid N2, and stored at −80 °C.

#### TadA:

Protein was purified as described above for TadA-GFPnb.

The identity of purified proteins was confirmed using standard mass spectrometry analyses in the Proteomics and Metabolomics Shared Resource at Fred Hutch.

### Antibody

GFP antibody (HtzGFP-19F7) was acquired from the Memorial Sloan Kettering Cancer Center.

### *In vitro* transcription

Synthetic DNA was purchased from IDT that encoded a T7 promoter (TAATACGACTCACTATAGG), the human β-globin 5′ UTR, a nanoLuciferase ORF, and the human β-globin 3′ UTR, with a boxB motif (GCCCTGAAGAAGGGC) either inserted or not. This was amplified with Phusion High-Fidelity DNA polymerase (ThermoFisher, cat # F530S) and primers CPL_184 and CPL_262. The PCR product was purified using a PureLink Quick PCR Purification Kit (Invitrogen, cat # K310002). After amplification, the RNAs were *in vitro* transcribed using a MEGAscript T7 Transcription Kit (Thermofisher, cat # AM1334) for 3 hours at 37 °C and treated with Turbo DNase for 15 minutes at 37 °C. Transcribed RNA was purified via a GeneJET RNA Purification kit (ThermoScientific, cat # K7032), followed by Micro Bio-Spin P-6 Gel Columns (Bio-Rad, cat # 7326221) equilibrated 3x with 200 uL of water. The purity of RNA was determined by agarose gel electrophoresis and quantified by Nanodrop.

### Low throughput RNA editing assay

The reporter mRNAs (125 nM each, containing either boxB or not) were refolded in water by heating to 95 °C for 2 minutes and then cooled slowly to RT. The refolded mRNAs were added to a reaction mixture containing TadA buffer (50 mM Tris-HCl pH 7.5, 25 mM KCl, 2 mM MgCl2). After incubating at 37 °C for 5 minutes, the indicated TadA recombinant protein (250 nM) was added. For nanobody and antibody recruitment, 200 nM and 100 nM of each reporter mRNA was used, respectively. Reactions were incubated at 37 °C for 2 hours. RNA was purified via a GeneJET RNA purification kit and quantified by Nanodrop. Purified RNA (375 ng) and DNA primer CPL_372 (1 *μ*M) were incubated together at 65 °C for 5 minutes and annealed on ice. RNA was reverse transcribed using Maxima RT (ThermoFisher, cat # EP0742) at 50 °C for 30 minutes followed by 85 °C for 5 minutes. The resulting cDNA was amplified by PCR as described above using primers CPL_373 and CPL_374. The PCR products were purified and analyzed by nanopore sequencing (Plasmidsaurus). FASTQ files containing basecalled nanopore reads were sorted into populations that either contained the boxB element or did not. In both populations, the 40 nucleotides immediately 3′ of the boxB motif insertion were compared against the non-edited reference sequence to identify adenosine to guanosine substitutions. Sequences with inserts or deletions were discarded. To determine the reported 95% confidence intervals, a bootstrapping analysis using rflip() from the mosaic package in R was used, with 100,000 iterations.

### BoxB reporter oligo library design

Oligos for cloning the *in vitro* reporter pool were designed using a custom R script design_invitro_oligo_pool.R to generate a comprehensive library of sequence variants. Each oligo was composed of a 5′ T7 promoter (TAATACGACTCACTATAGG), a forward handle (TGGCTTCGTTGTTGTGCT), a variable spacer region (TTTGTGTTCTCTTGTTCGTTCTGGTTCGTT), a recorder region (TAGAATTACACCATAAT), and the boxB stem loop (GGGCCCTGAAGAAGGGCCC), with additional short buffer sequences flanking the variable regions. Barcode sequences devoid of A and separated by a Hamming distance of 2 were used to uniquely tag every oligo. To generate spacer variants, the full-length spacer sequence was truncated in two-nucleotide increments, creating a set of fragments; for each truncation, the spacer was split into 5′ and 3′ segments, and the fixed recorder region was inserted between these fragments to form a “spacer target” sequence. For randomizing the recorder region, all non-A nucleotides within the recorder were independently randomized in groups of 5 nucleotides to yield two distinct sets of target sequences. Each randomized spacer target was then incorporated into two oligo orientations by appending the boxB stem loop together with its buffer on either the 5′ or the 3′ side. The boxB stem loop was randomized in 3 or nt increments. The final oligo sequences are assembled by concatenating the T7 promoter, the forward handle, the designed variable region (incorporating spacer, recorder region, and boxB stem loops with their buffers), the barcode buffer with the assigned barcode, and a reverse transcription handle (GCTGGCTTCTGTTCCGTTTG). This oligo pool was ordered from IDT as oPool oAS2176. See Supplementary Table 4 for the full list.

Oligos for the *in vivo* reporter pool with randomized boxB stem loops were designed as above and ordered from IDT as oPool oRB262. See Supplementary Table 5 for full list.

Oligos for the λN site saturation mutagenesis library were designed by replacing each codon in the λN ORF by NNN. The oligos were ordered from IDT as oPool oRB275. See Supplementary Table 6 for full list.

### High throughput *in vitro* RNA editing assay

CPL_303 (10 *μ*M) was pre-annealed to the oligo pool oAS2176 (10 *μ*M) in annealing buffer (20 mM Hepes pH 7.5, 100 mM KCl, and 2 mM MgCl2) by heating to 70 °C then cooling by 0.2 °C/s. The pool was transcribed and treated with DNase as described above. RNA was purified by phenol:chloroform extraction and ethanol precipitation.

The RNA libraries (250 nM total concentration) were added to TadA buffer along with any non-TadA fused protein (125, 250, or 500 nM). After incubating at 37 °C for 5 minutes, the indicated TadA recombinant protein was added equimolar to any non-TadA protein present. If the reaction contained the anti-GFP antibody, it was instead incubated for 1 hour at room temperature before TadA recombinant protein addition as previously described (Xiao et al. 2024a). The reactions were incubated at either 37 °C for 0.5, 1, or 2 hours, or 25 °C or 13 °C for 2 hours. RNA was purified by phenol:chloroform extraction and ethanol precipitation.

### Cell culture

HEK293T cells were cultured in Dulbecco’s modified Eagle medium (DMEM 1X, with 4.5 g/L D-glucose, + L-glutamine, − sodium pyruvate, Gibco 11965-092) supplemented with 10% FBS (Thermo 26140079) and passaged using 0.25% trypsin in EDTA (Gibco 25200-056). Cells were grown at 37 °C in 5% CO2. Cell lines were confirmed to be free of mycoplasma contamination.

### Integration of plasmid libraries into landing pad cell lines

hsAS126.3 (HEK293T attP* Cas9) cells (Nugent et al. 2024) were seeded to 80% confluency in one 10 cm dish per library. 9.6 μg of attB* -containing reporter library plasmid (pAS475, pAS476, and pAS517) and 2.4 μg of Bxb1 expression vector (pAS344) were transfected per 10 cm dish using FuGENE HD reagent (Promega). Each library was transfected into a single 10 cm dish then expanded into 15 cm dishes 48 hours post-transfection. Cells were selected with 2 μg/ml puromycin, added 72 hours post-transfection. Puromycin selection was ended after 8 days, and cell pools were contracted back into a 10 cm dish. 24h after ending puromycin selection, 2 μg/ml doxycycline was added to induce TadA and boxB library reporter expression.

### Library mRNA extraction

Library mRNA was harvested after 72 hr of doxycycline induction of TadA and boxB reporters from one 50-75% confluent 10 cm dish. Each 10 cm dish was treated with 1 ml .025% Trypsin, and neutralized with 5 mL DMEM media. Cells were pelleted from 1/3 of this cell suspension and resuspended in 1 ml Trizol reagent (Thermo). Total RNA from these lysates was then extracted using the Direct-zol RNA Miniprep kit (Zymo) following the manufacturer’s protocol.

### High throughput sequencing of boxB reporters

2.3-7 μg of total RNA from *in vivo* libraries or 25-200 ng RNA from each *in vitro* enzymatic reaction was reverse transcribed into cDNA using Maxima H Minus reverse transcriptase (Thermo) and RT primer oRB213 which also contains a 7 nt UMI. A 20-50 μl PCR was performed using Phusion polymerase (Thermo) for 6-22 cycles with cDNA template comprising 1/20th of the final volume, and with oPN776 as the reverse primer. Indexed forward primers were used to enable pooled sequencing of all samples (one of oRB218-oRB225 or oRB287-oRB302). All PCR reactions generated a 192 bp amplicon that was cut out from a 2% agarose gel and cleaned using the Zymoclean Gel DNA Recovery Kit (Zymo). Libraries were sequenced on an Illumina NextSeq 2000 using custom sequencing primers. Custom primers were oRB214 for Read 1 (79 bp read), oRB215 for Read 2 (7bp read), and oRB217 for indexing (7bp read).

### Computational analyses

Pre-processing steps for high-throughput sequencing were implemented as Snakemake (Köster and Rahmann 2012) workflows run within Singularity containers on an HPC cluster. All container images used in this study are publicly available as Docker images at https://github.com/orgs/rasilab/packages. Python (v3.9.15) and R (v4.2.2) programming languages were used for all analyses unless mentioned otherwise.

### Edited base counting for each boxB reporter variable region insert

The raw data from boxB reporter sequencing are in FASTQ format. The boxB reporter oligo pool sequences was used to create a reference annotations file called barcode_annotations.csv containing 10-nt barcodes identifying the locations of the A-Rich recorder region and variable region within the reporter sequence read. The 10 nt barcode of each read was extracted and used to assign the entire read to an individual FASTQ file for each barcode in the split_by_barcode.awk script. The calculate_summary_stats.ipynb script then filtered reads to determine whether invariant sequences upstream and downstream of the A-rich reporter region match those documented in barcode_annotations.csv for that barcode. If a read passed the above filters, the A-rich recorder region, variable insert region, and UMI from each read was extracted according the start and length parameters for that barcode file referenced in barcode_annotations.csv. Only the first instance of each UMI was tallied.

For each unique combination of variable region, the total UMI count was tallied, as well as the the number of A,C,T and G reads for each of the 8 adenosine sites within the A-rich target region. Additionally, the number of reads with 0,1,2…8 total A,T,C and Gs were tallied for unique insert. The final list of insert, UMI and recorder region adenosine counts was printed as a .csv file for each boxB reporter barcode. These .csv files for each boxB reporter barcode were concatenated into one .csv table per condition for subseqeunce analysis using the combine_barcode_summary_stats.ipynb script.

### Statistical Methods

For comparing GNRNA BoxB motifs: BoxB sequence variants were filtered to include only those for which >200 UMIs were detected, and maintained the closing U-A base pair at boxB positions 7 & 13.

Stem variants: Mean percent of reads with one or more adenine-to-guanine transitions observed in the recorder region was calculated for each stem loop variant across n=4 technical replicates. Each stem variant was assigned to percentile of free energy distribution based on Gibbs free energy calculated by RNAFold (see Figure 2I), such that each distribution represents n=50 or 51 stem variants.

DMS: The bootstrapped mean percentage of reads with 1+ base edits was calculated for each peptide variant and normalized to the bootstrapped mean for wild-type λN.

## Supplementary Material

Supplement 1

2

## Data Availability

All high-throughput sequencing data generated in this study are available at the NCBI Sequence Read Archive (SRA) under BioProject PRJNA1255650. All other data and programming code associated with this manuscript are publicly available at https://github.com/rasilab/bakker_2025.
