# Supplementary material for: Deaminase-based RNA recording enables high throughput mutational profiling of protein-RNA interactions
Source: bioRxiv. 2025 Apr 25:2025.04.11.648485. Originally published 2025 Apr 12. Preprint. [Version 2] doi: 10.1101/2025.04.11.648485 (PMC12027372; doi:10.1101/2025.04.11.648485)
Supplement: 2 [file NIHPP2025.04.11.648485v2-supplement-2.pdf]

bioRxiv preprint doi: https://doi.org/10.1101/2025.04.11.648485; this version posted April 25, 2025. The copyright holder for this preprint (which was not certified by peer review) is the author/funder, who has granted bioRxiv a license to display the preprint in perpetuity. It is made available under aCC-BY 4.0 International license.

Supplementary Figures

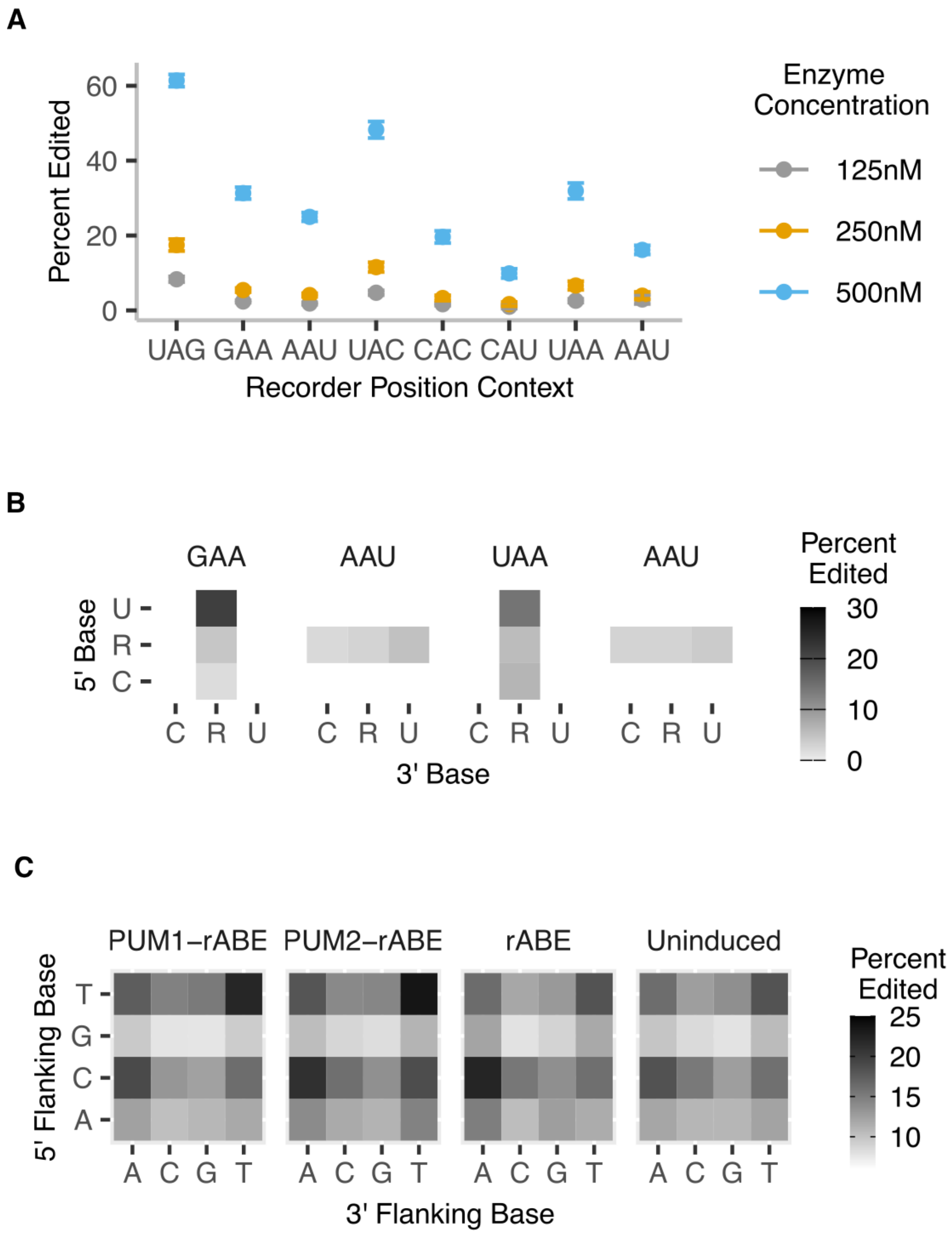

Supplementary Figure 1: Analysis of TadA-λN editing.
A. Mean editing efficiency at different adenines within the recorder region for different concentrations of TadA-λN. Error bars denote standard error over 24 technical replicates.
B. Mean editing efficiency as a function of the nucleotide flanking the edited adenine. R represents G and A nucleotides, which were tallied together since we cannot resolve edited As from unedited Gs. Mean is calculated over 30 technical replicates.
C. Analysis of editing context dependence using RNA-Seq data from Lin et al (Lin et al. 2023). Heatmaps indicate mean percent of reads edited for all sites with the indicated 5′ and 3′ flanking bases. Only sites with at least 1 edited read and >10 total reads were included in this analysis.

bioRxiv preprint doi: https://doi.org/10.1101/2025.04.11.648485; this version posted April 25, 2025. The copyright holder for this preprint (which was not certified by peer review) is the author/funder, who has granted bioRxiv a license to display the preprint in perpetuity. It is made available under aCC-BY 4.0 International license.

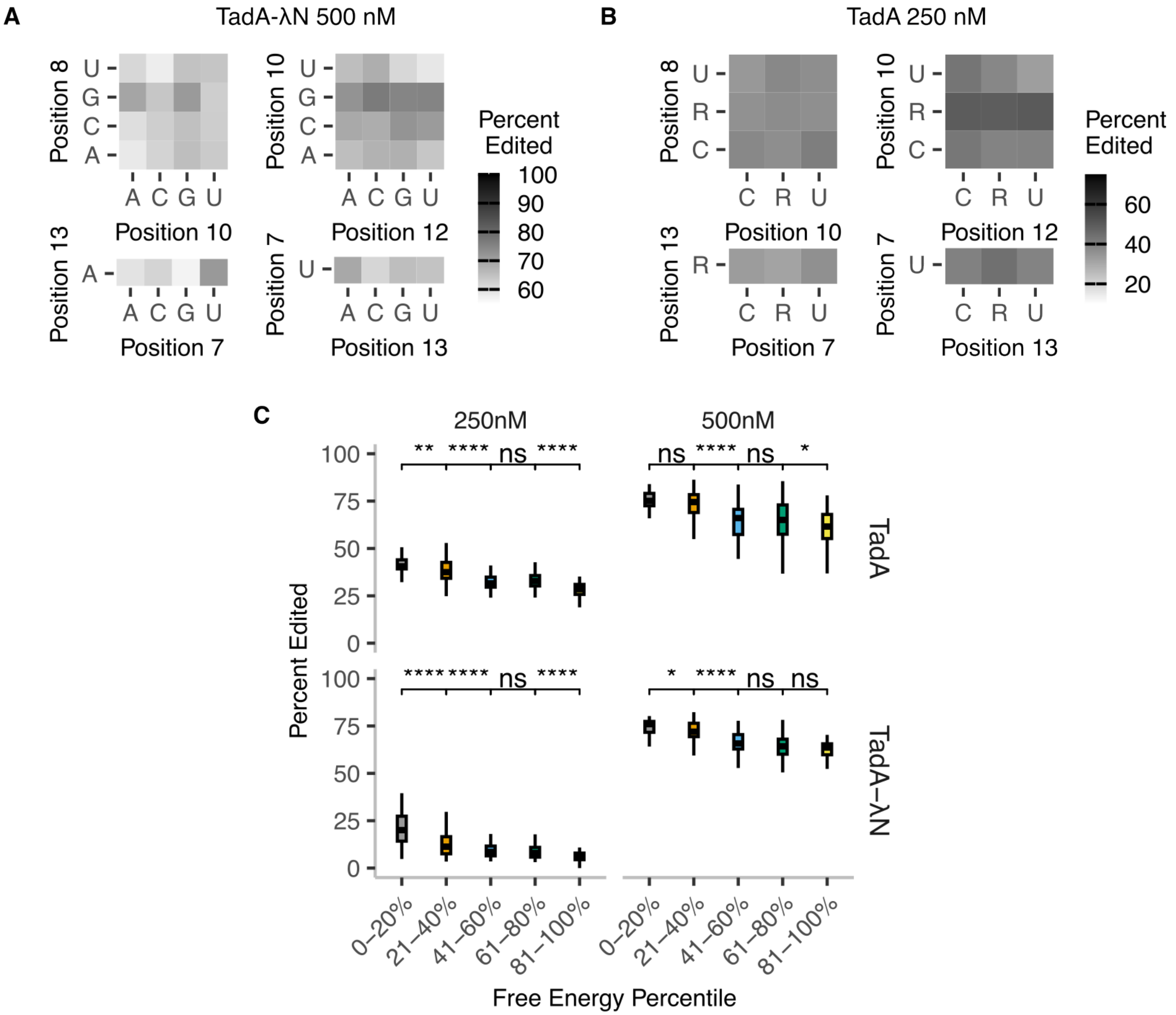

Supplementary Figure 2: Analysis of TadA8.20 editing in nonspecific contexts.
A. Mean editing efficiency as a function of nucleotide identity at location 8 and 10 (top-left heatmap), 10 and 12 (bottom-right) and base 7 and 13 (bottom heatmaps) of the boxB loop for TadA-λN fusion at 500nM Scales identical to those in Figure 3D-E for ease of comparison.
B. Mean editing efficiency as a function of nucleotide identity at location 8 and 10 (top-left heatmap), 10 and 12 (bottom-right) and base 7 and 13 (bottom heatmaps) of the boxB loop for TadA alone Scales identical to those in Figure 3D-E for ease of comparison. R represents G and A bases, which cannot be resolved due to the high rate of TadA editing in the boxB loop (Figure 2C).
C Mean editing efficiency of boxB stem variants for TadA-λN and TadA alone at 250nM and 500 nM. Free energy intervals are identical to those indicated in Figure 2J x-axis. Box plots indicate median and inter-quartile ranges. P-values were calculated using two-sided Wilcoxon test. **** $p < 0.0001$, *** $p<0.001$, ** $p<0.01$, * $p<0.05$, n.s $p > 0.05$.

bioRxiv preprint doi: https://doi.org/10.1101/2025.04.11.648485; this version posted April 25, 2025. The copyright holder for this preprint (which was not certified by peer review) is the author/funder, who has granted bioRxiv a license to display the preprint in perpetuity. It is made available under aCC-BY 4.0 International license.

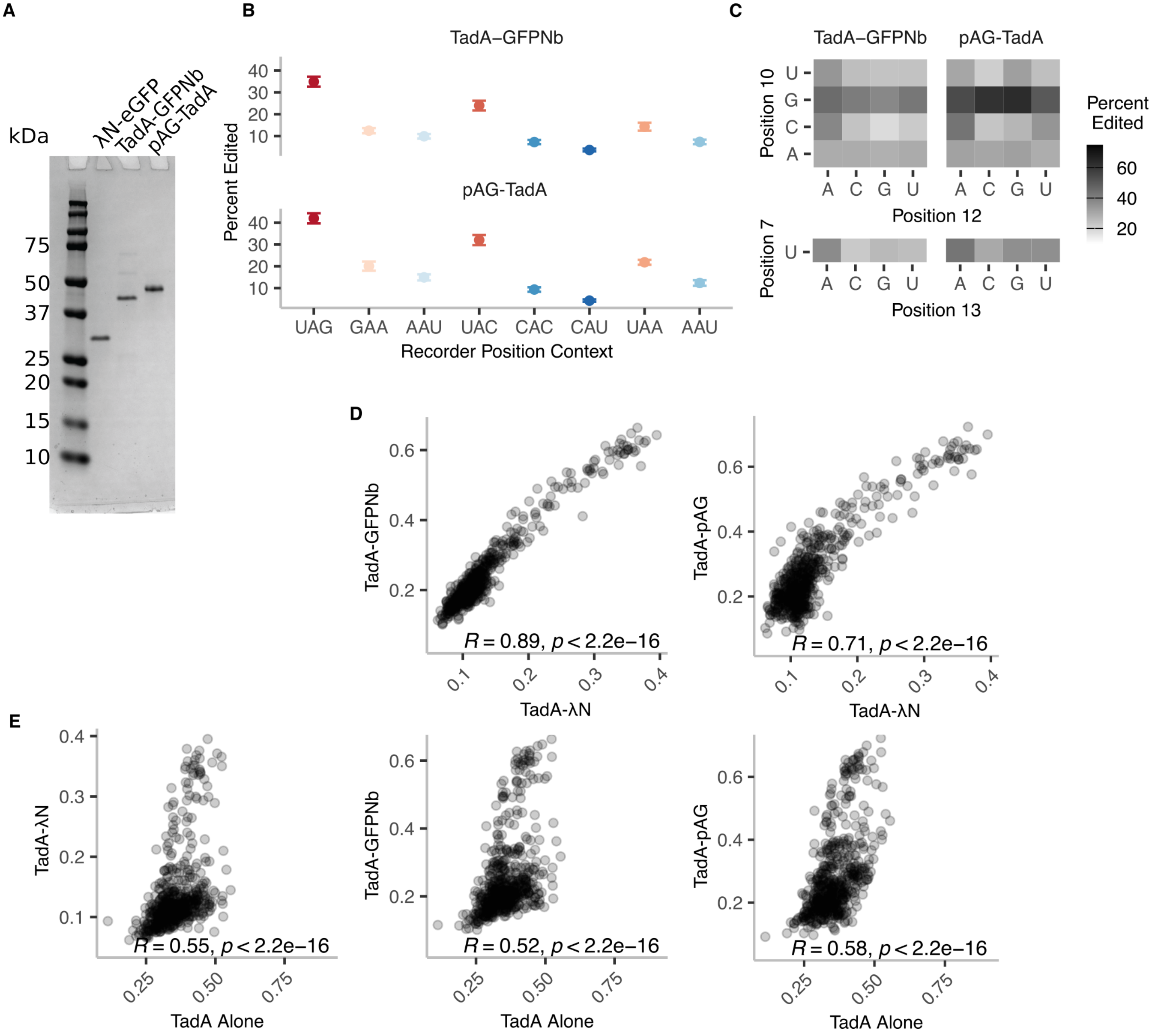

Supplementary Figure 3: Analysis of TadA recruitment strategies.

A. SDS-PAGE analysis of purified λN-EGFP, TadA-GFPNb and pAG-TadA. Proteins visualized with Coomassie stain.

B. Quantification of editing of individual adenines within the recorder region for TadA-GFPNb (top panel) and pAG-TadA (bottom panel). Each point represents mean percentage of reads with an adenine-to-guanine transition observed at that position. The mean was calculated from each of n=24 independent reporter libraries where reporter sequence was constant. Error bars represent standard error of the mean.

C. Mean editing efficiency as a function of nucleotide identity at location 10 and 12 (top heatmap) and 13 (bottom heatmaps) of the boxB loop for TadA alone. Scales identical to those in Figure 5E for comparison.

D. Comparison of editing efficiency between TadA-GFPNb and TadA-λN (left) and pAG-TadA and TadA-λN (right). R is Spearman correlation coefficient.

E. Comparison of editing efficiency between TadA-λN (left), TadA-GFPNb (middle), pAG-TadA (right) and TadA8.20 alone. R is Spearman correlation coefficient.

bioRxiv preprint doi: https://doi.org/10.1101/2025.04.11.648485; this version posted April 25, 2025. The copyright holder for this preprint (which was not certified by peer review) is the author/funder, who has granted bioRxiv a license to display the preprint in perpetuity. It is made available under aCC-BY 4.0 International license.

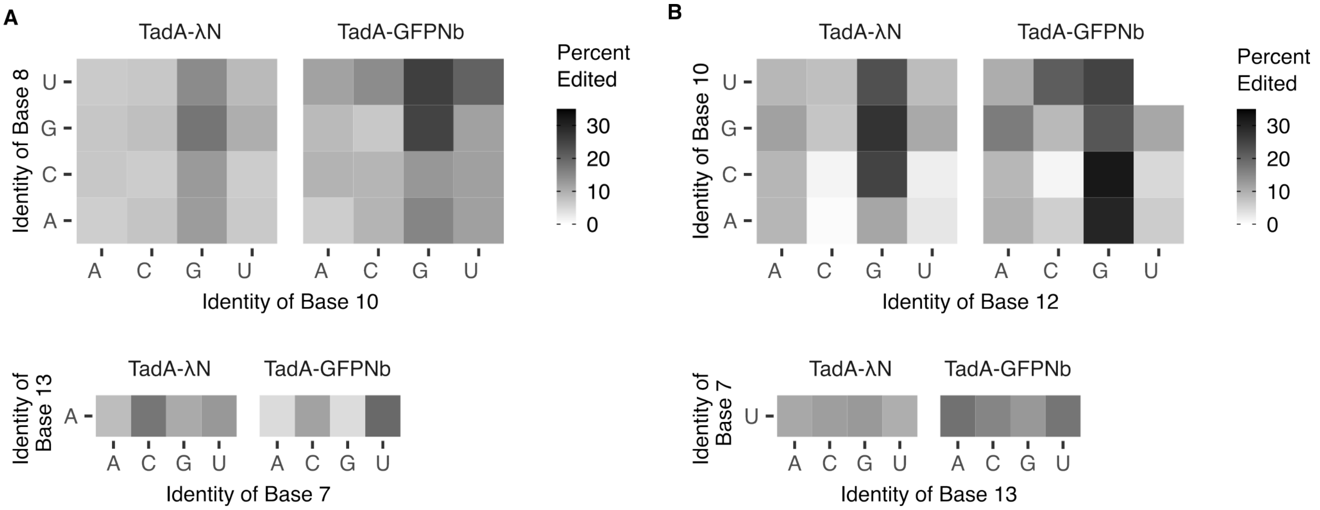

Supplementary Figure 4: Analysis of *in vivo* TadA-λN and TadA-GFPnb editing.
A. Mean editing efficiency as a function of nucleotide identity at location 8 and 10 (top heatmap) and 7 (bottom heatmaps) of the boxB loop for TadA alone. Scales identical to those in Figure 6 for comparison.
B. Mean editing efficiency as a function of nucleotide identity at location 10 and 12 (top heatmap) and 13 (bottom heatmaps) of the boxB loop for TadA alone. Scales identical to those in Figure 6 for comparison.

bioRxiv preprint doi: https://doi.org/10.1101/2025.04.11.648485; this version posted April 25, 2025. The copyright holder for this preprint (which was not certified by peer review) is the author/funder, who has granted bioRxiv a license to display the preprint in perpetuity. It is made available under aCC-BY 4.0 International license.

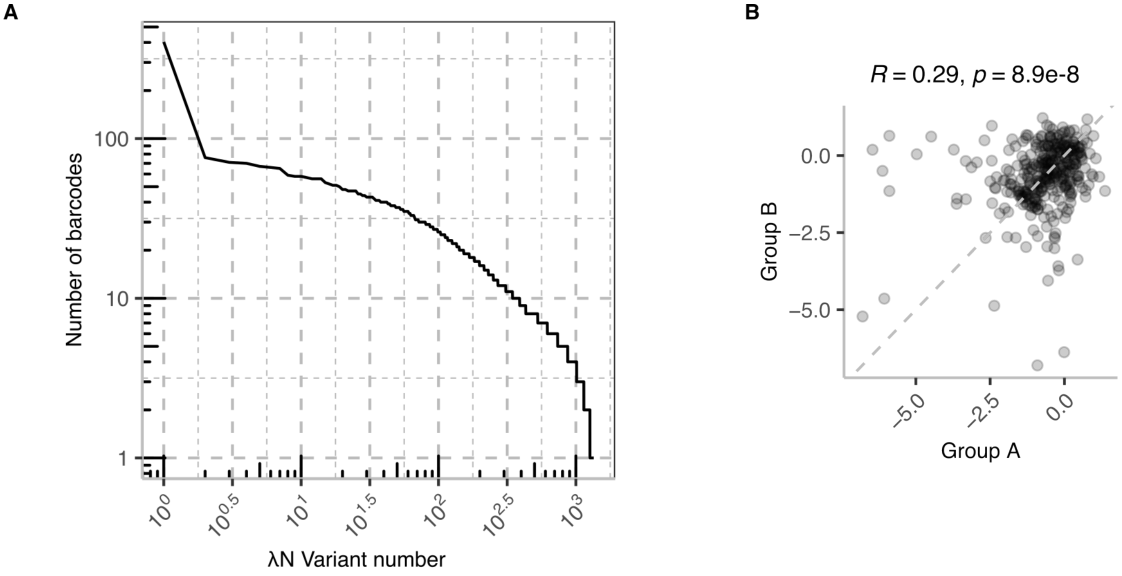

Supplementary Figure 5: Analysis of λN mutational scanning.
A. Linked barcodes per unique λN sequence variant. Unique 20nt barcodes were assigned to λN sequence variants via deep sequencing of the plasmid pool. Sequence variants were arranged by number of barcodes assigned and given a number, plotted on the x-axis. The number of linked barcodes is plotted on the y-axis. The “Wild-type” λN occurred at 22x times frequency in the plasmid pool and thus has a large number of barcodes assigned to it compared to all other sequences.
B. Correlation between barcode sets. For each λN amino acid variant, individual linked barcodes were randomly partitioned into two sets, (or to within a barcode for odd number of detected barcodes). R refers to Spearman correlation coefficient between barcode groups.
